# Definitive hematopoietic stem/progenitor cells from human embryonic stem cells through serum/feeder-free organoid-induced differentiation

**DOI:** 10.1186/s13287-020-02019-5

**Published:** 2020-11-24

**Authors:** Selami Demirci, Juan J. Haro-Mora, Alexis Leonard, Claire Drysdale, Daniela Malide, Keyvan Keyvanfar, Khaled Essawi, Raul Vizcardo, Naritaka Tamaoki, Nicholas P. Restifo, John F. Tisdale, Naoya Uchida

**Affiliations:** 1 Sickle Cell Branch, National Heart Lung and Blood Institute (NHLBI)/National Institute of Diabetes and Digestive and Kidney Diseases, National Institutes of Health (NIH), 9000 Rockville Pike, Bldg. 10, 9N112, Bethesda, MD 20892 USA; 2 Light Microscopy Core Facility, NHLBI, NIH, Bethesda, MD USA; 3 Clinical Flow Core Facility, NHLBI, NIH, Bethesda, MD USA; 4 National Cancer Institute, Center for Cancer Research, NIH, Bethesda, MD USA

**Keywords:** iPSCs, Hemogenic endothelium, HSCs, T cell differentiation, β-Globin, Definitive hematopoiesis

## Abstract

**Background:**

Ex vivo production of hematopoietic stem/precursor cells (HSPCs) represents a promising versatile approach for blood disorders.

**Methods:**

To derive definitive HSPCs from human embryonic stem cells (ESCs), we differentiated mesodermally specified embryoid bodies (EBs) on gelatin-coated plates in serum/feeder-free conditions.

**Results:**

Seven-day EB maturation followed by an 8-day differentiation period on OP9 cells provided the highest number of definitive (CD34+ CD235a−, 69%, *p* < 0.01) and lowest number of primitive (CD34− CD235a+, 1.55%, *p* < 0.01) precursor cells along with the highest colony-forming units (149.8 ± 11.6, *p* < 0.01) in feeder-free conditions. Maximal HSPC fraction (CD34+ CD38− CD45RA− CD49f+ CD90+) was 7.6–8.9% after 10 days of hematopoietic differentiation with 14.5% adult β-globin expression following RBC differentiation. Myeloid and erythroid colonies were restricted strictly to the CD34+ CD43+ fraction (370.5 ± 65.7, *p* < 0.001), while the CD34− CD43+ fraction produced only a small number of colonies (21.6 ± 11.9). In addition, we differentiated the CD34+ CD43+ cells towards T-lymphocytes using the OP9/DLL1 co-culture system demonstrating double-positive T cells (CD4+ CD8+) with CD3+ expression displaying a broad T cell receptor (TCR) repertoire. Confocal imaging of organoid-like structures revealed a close association of CD31+ cells with CD34+ and CD43+ cells, suggesting a potential emergence of HSPCs through endothelial to hematopoietic transition. Furthermore, fluorescently labeled organoids exhibited the emergence of spherical non-attached cells from rare progenitors at the border of the organoid center.

**Conclusions:**

In summary, definitive HSPCs can be derived from ESCs through a dynamic cellular process from an organoid-like structure, where erythroid progeny are capable of producing adult hemoglobin and lymphoid progeny shows a diverse TCR repertoire.

## Background

Hematopoietic stem cells (HSCs) give rise to all blood cells, provide a lifetime supply of mature blood cells, and can reconstitute the whole hematopoietic system of a conditioned recipient after infusion. However, there is scarce availability of human leukocyte antigen (HLA)-matched donors and ex vivo cultured HSCs display a reduced engraftment capacity; therefore, alternative HSC sources and/or culture conditions that maintain stem cell characteristics are preferred [1–3]. In theory, embryonic stem cell (ESC)/induced pluripotent stem cell (iPSC) cultures could enable unlimited HSC production. Subcutaneous transplantation of ESCs/iPSCs are capable of long-term engraftment and blood cell production in serially transplanted mice [4, 5], indicating the biological sufficiency of pluripotent stem cells for HSC production under the right conditions. To date, generating true HSCs capable of engraftment in immunocompromised mice requires engineering with specific sets of transcription factors [6, 7]. However, of those approaches, *MLL-AF4*-engineered iPSCs displayed leukemic transformation during long-term follow-up [7], and dysregulation of the HOX pathway is strongly associated with leukemia progression [8]. Thus, developing safer ex vivo HSC generating protocols, preferably without deleterious transcription factor manipulation, is of importance to international research and for translation towards clinical applicability.

To establish a successful and efficient ex vivo HSC differentiation method from ESCs/iPSCs, it is vital to unravel developmental hematopoiesis, which is comprised of multiple, partially overlapping but spatiotemporally separated waves: (i) a primitive yolk sac wave generating macrophages, megakaryocytes, and primitive red blood cells (RBCs) with mainly ε-globin expression, (ii) a definitive yolk sac wave producing erythro-myeloid and lympho-myeloid progenitors, and (iii) engraftable HSC generation in a third wave from hemogenic endothelium (HE) cells emerging from the aorta gonad-mesonephros (AGM) region through a process called endothelial-to-hematopoietic transition (EHT) (reviewed in [9]). Early studies using ESC cultures could only recapitulate yolk sac primitive and partial yolk sac definitive hematopoiesis that could generate hematopoietic stem/progenitor cells (HSPCs) without the ability to engraft [10, 11]. Elucidating crucial signaling pathways and globin switching mechanisms led to more sophisticated protocols that can produce HSPCs with a more definitive phenotype, yet engraftment capacity is still lacking with these methods [12–14].

Applying advanced imaging approaches to embryos has revealed vital processes of developmental hematopoiesis including the precise timing of HSC emergence from aortic endothelium [15] and that life-long hematopoiesis is supported by a small number of HSCs [16]. Here, we optimize a two-step protocol for hematopoietic differentiation of human ESCs to derive definitive HSPCs capable of producing RBCs with adult globin (β-globin and γ-globin) and T-lymphocytes with a broad T cell receptor (TCR) repertoire. Moreover, imaging of antibody-stained and fluorescently tagged organoids provided insights into the emergence of multipotent definitive HSPCs from rare progenitors that closely associate with endothelial cells. These results help the establishment of clinical-grade ex vivo protocols for the generation of definitive HSPCs for transfusion purposes and/or engraftable HSC for transplantation studies.

## Materials and methods

### Cell culture conditions

Human ESC line (H1, passage 46–65, WiCell, Madison, WI) was cultured on Matrigel Growth Factor Reduced Basement Membrane Matrix (Corning, New York, NY)-coated 6-well plates (Corning) using mTeSR1 media (Stem Cell Technologies, Vancouver, Canada) at 37 °C and 5% CO_2_. The media was changed daily (except weekends) and cells were passaged every 7 days using an EDTA protocol [17].

Mouse bone marrow stromal cells (OP9 cells, CRL-2749, ATCC, Manassas, VA) were grown in alpha minimum essential medium without ribonucleosides and deoxyribonucleosides and with 2.2 g/L sodium bicarbonate containing 20% fetal bovine serum (FBS, Thermo Fisher Scientific, New York, NY). Cells were passaged at 70% confluency and irradiated at 50 Gy before use. HeLa cells (CCL-2, ATCC) were maintained in Dulbecco’s modified Eagle medium (DMEM) containing 20% FBS.

### Hematopoietic differentiation of ESC

HSPC containing organoid-like structures were generated using a two-step protocol by coupling embryoid body (EB) formation and organoid differentiation of EBs on irradiated OP9, gelatin, or matrigel-coated cell culture plates. Differentiation media compositions are given in Table 1. In short, 50% confluent ESCs were treated with accutase solution (Sigma-Aldrich, St. Louis, MO) for 5 min at 37 °C and 5% CO_2_. Single cells (4 × 10^3^/EB) were suspended in serum-free EB media (SFM) containing 1× rock inhibitor (Stem Cell Technologies), 10 ng/ml bone morphogenic protein-4 (BMP4, R&D Systems, Minneapolis, MN), and 10 ng/ml basic fibroblast growth factor (bFGF, Pepro Tech, Rockey hill, NJ). Media (50 μl) containing single ESCs was dispersed into low-attachment, round-bottomed 96-well plates (Corning, Acton, MA). The plates were centrifuged at 125×*g* for 3 min and incubated at 37 °C and 5% CO_2_. At day 2, 50 μl of SFM containing 10 ng/ml BMP-4, 10 ng/ml bFGF, 100 ng/ml stem cell factor (SCF, R&D Systems), and 20 ng/ml vascular endothelial growth factor (VEGF, Pepro Tech) was added to each well. The media was changed every 2 days with SFM containing 10 ng/ml BMP-4, 10 ng/ml bFGF, 50 ng/ml SCF, and 10 ng/ml VEGF during EB maturation period.

**Table 1:** Hematopoietic stem/progenitor cell (HSPC) differentiation media composition

Embryoid body (EB) serum-free media [18]		Organoid differentiation [12]	
IMDM (Sigma-Aldrich)	243 ml	IMDM	390 ml
Ham’s F12 (Sigma-Aldrich)	243 ml	Knockout serum replacement (Thermo Fisher)	100 ml
BSA (Roche)	2.5 g	ITS (100×)	5 ml
Insulin-Transferrin-Selenium (ITS, 100×, Sigma-Aldrich)	5 ml	Ascorbic acid	25 mg
Lipid mixture (100×, Thermo Fisher, Waltham, MA)	5 ml	L-glutamine (100×)	5 ml
Ascorbic acid (Sigma-Aldrich)	25 mg	1-thioglycerol	19 μl
L-glutamine (100×, Thermo Fisher)	5 ml		
1-thioglycerol (Sigma-Aldrich)	19 μl		

In the second phase, EBs were collected and resuspended in organoid differentiation media (Table 1) containing 20 ng/ml VEGF and transferred to gelatin, matrigel, or irradiated OP9-coated cell culture plates (1.5 EBs/cm^2^). The media was refreshed every 2 days. At the end of the differentiation period, organoid-like structures were treated with 5 ml of collagenase type IV (50 U/ml) (Thermo Fisher Scientific) followed by 0.05% Trypsin-EDTA (Thermo Fisher Scientific) treatment for 30 min each at 37 °C. Dissociated cells were filtered through 70-μm cell strainers and washed with phosphate buffer solution (PBS) to be used in subsequent studies.

### Colony-forming unit (CFU) assay

Single cells from organoid-like structures were resuspended in Iscove’s modified Dulbecco’s medium (IMDM, Sigma-Aldrich) containing 2% FBS at a concentration of 1 × 10^6^ cells/ml. One milliliters of MethoCult H4434 Classic (Stem Cell Technologies) was mixed with 100 μl of cell suspension (1 × 10^5^ cells) in falcon tubes and the tube was vortexed thoroughly for 30 s. Air bubble-removed mixtures (1.1 ml/plate) were transferred to 35-mm Petri plates (Corning) and incubated for 14 days at 37 °C and 5% CO_2_. The plates were visually scored for CFU-GM, CFU-E/BFU-E, and CFU-GEMM.

### RBC differentiation

To differentiate ESC-derived HSPCs towards erythrocytes, cells were first incubated in transfer media consisting of organoid differentiation media with 50 ng/ml FMS-like tyrosine kinase 3 ligand (FLT-3L, R&D systems), 50 ng/ml thrombopoietin (TPO, R&D systems), 5 ng/ml interleukin-3 (IL-3; R&D systems), 50 ng/ml SCF, 5 U/mL erythropoietin (EPO, AMGEN, Thousand Oaks, CA), and 10 ng/ml BMP-4 on irradiated OP9 cells to eliminate adherent cells. After 2 days, non-attached cells were transferred onto fresh irradiated OP9 cell-coated plates in proliferation media consisting of IMDM media with 10 ng/ml SCF, 1 ng/ml IL-3, 2 U/ml EPO, 1 μM estradiol (Pfizer, New York, NY), 1 μM dexamethasone (VETone, Boise, ID), and 20% FBS for 6 days. For maturation phase of RBC differentiation, the media was replaced with IMDM containing 2% bovine serum albumin (BSA, Roche, Indianapolis, IN), 0.56 mg/mL transferrin (Sigma-Aldrich), 2 mM L-glutamine, 2 U/ml EPO, 10 ng/ml insulin (Lilly, Indianapolis, IN), and 20% FBS. The media was changed every 2–3 days for a total of 15 days of RBC differentiation.

### Hemoglobin (Hb) electrophoresis

Different Hb types in RBCs (1–3 × 10^6^ cells) derived from ESC-HSPCs (at day 15 of RBC differentiation) were determined by electrophoresis using cellulose acetate membranes and alkaline buffer solution according to the manufacturer’s instructions (Helena Laboratories, Beaumont, TX).

### Reverse-phase high-performance liquid chromatography (RP-HPLC)

To determine the relative globin chain expressions in differentiated RBCs (0.5–1.5 × 10^6^ cells), RP-HPLC was conducted as previously described [19]. Briefly, pelleted cells were washed three times with PBS and lysed in 100 μl of HPLC-grade water (Sigma-Aldrich) by vortexing 30 s (3×). After centrifugation at 16,000×*g* for 10 min, 90 μl of supernatant was transferred to a new Eppendorf tube and 10 μl of Tris (2-carboxyethyl) phosphine hydrochloride (TCEP; 100 mmol; Thermo Fisher Scientific) was added followed by 5-min incubation at room temperature (RT). After adding 85 μl of 0.1% trifluoroacetic acid (TFA)/32% acetonitrile and vortexing briefly, the mixture was centrifuged at 16,000×*g* for 5 min. Supernatant (10–40 μl) was analyzed by an Agilent 1100 HPLC (Agilent Technologies, Santa Clara, CA) equipped with a reverse-phase column, Aeris 3.6 μm Widepore C4 200 (250 × 4.6 mm; Phenomenex, Torrance, CA) using 0.12% Trifluoroacetic acid (TFA, Sigma-Aldrich) in water as solvent A, and 0.08% TFA in acetonitrile (Sigma-Aldrich) at a 0.7 mL/min flow rate for 50 min. The globin chain peaks were detected at 215 nm.

### Flow cytometry

Cell surface marker expression analysis was conducted by flow cytometry using either a FACSCalibur (for 3-color panels, Becton Dickinson, East Rutherford, NJ) or a BD FACSCanto flow cytometer (for 6-color panels) after fluorescent antibody labeling. All antibodies were provided by BD Biosciences (San Jose, CA) as follows: CD31-APC Cy7 (clone WM59), CD31-FITC (WM59), CD34-PerCP Cy5.5 (clone 8G12), CD34-PE (clone 563), CD34-FITC (clone 581), CD38-APC (Clone HIT2), CD43-APC (clone 1G10), CD45-APC (Clone HI30), CD45-APC Cy7 (Clone 2D1), CD45RA-APC H7 (Clone HI100), CD49f-PE (Clone GoH3), CD73-APC (clone M-A712), CD90-PE Cy7 (Clone 5E10), CD144-FITC (Clone 55-7H1), CD235a-PE Cy7 (clone GA-R2), CD235a-PE (clone GA-R2), and DLL4-PE (Clone MHD4-46). Sorting experiments were performed using a BD FACSAria II instrument.

### T cell differentiation

Generation of T cells from human ESCs was performed using a slightly modified OP9/DLL1 stromal cell co-culture system [20]. Briefly, sorted CD34+ CD43+ cells from organoid-like structures at day 15 were plated in an OP9/DLL1 semi-confluent dish in OP9 medium containing IL-7 (5 ng/ml, R&D Systems), FLT-3L (5 ng/ml), and SCF (10 ng/ml). On day 3 of T cell differentiation, semi-adherent cells were collected and passaged into a new OP9/DLL1 dish. After this point, cell passaging was conducted every 7 days. From day 17, semi-adherent cells were passaged every 5 days and flow cytometry analysis was conducted at day 22 and day 27 using antibodies from BD Biosciences; CD3-Bv450 (clone UCHT1), CD4-PEcy7 (clone RPAT4), CD5-Bv605 (clone UCHT2), CD7-PE (clone MT701), and CD8-APC (clone RPA-T8). TCR-Vβ deep sequencing was performed by immunoSEQ (Adaptive Biotechnologies, Seattle, WA) on genomic DNA isolated from CD4+CD8+CD3+ cells on day 27 of T cell differentiation.

### Fluorescent labeling of ESCs

To have sustained fluorescent protein expression in human ESCs, cells were fluorescently labeled by AAVS1-targeted integration using CRISPR/Cas9 editing. To prepare donor plasmids carrying respective fluorescent protein (FP) sequences, GFP sequence in the pAAVS1P-iCAG.copGFP plasmid (AddGene #66577) was replaced with one of cerulean, EGFP, Venus, or tdTomato derived from AddGene plasmids #27338, #25917, #27340, and #27342, respectively. Then, accutase-treated ESCs are electroporated with ribonucleoprotein (RNP) consisting of chemically modified synthetic AAVS1 (CUCCCUCCCAGGAUCCUCUC) sgRNA (2′-*O*-methyl-3′-phosphorothioate modifications in the first and last three nucleotides) (600 nmol, Synthego Co., CA) and SpCas9 (200 pmol, University of California, Berkeley, CA) in 20 of P3 Primary Cell Nucleofector solution (V4XP-3032, Lonza, Basel, Switzerland) using Amaxa™ 4D-Nucleofector™ (Lonza). sgRNA and Cas9 were mixed and incubated at RT for 15 min to form RNP. Cells (2 × 10^5^ cells) suspended in P3 solution were added donor plasmids (300 pmol) carrying FP sequences (cerulean, EGFP, Venus, or tdTomato) and *AAVS1* homology arms (0.8 kb each side). Cells and plasmid mixture was combined with RNP and immediately transferred to respective transfection wells to electroporate with CB-150 program. Then, electroporated cells were transferred to matrigel-coated wells in mTESR1 media containing 1× rock inhibitor. After 2–3 days of culture, a small number of fluorescently positive cells were picked under microscopic evaluation and grown separately. At 50% confluency, accutase-treated cells were sorted for fluorescent positive cells. Sorted cells were plated in mTESR1 media containing rock inhibitor, and grown cells were cryopreserved for later use.

### Confocal and two-photon microscopy and image analysis

*Immunostaining of whole-mount organoid structures* was performed as previously described [21]. Briefly, whole-mount organoids were fixed in 4% paraformaldehyde (PFA) (Sigma-Aldrich) in PBS (Corning) for 1 h at RT, washed (3×) for 15 min with PBS, and then incubated in 10% donkey serum (EMD Millipore, Billerica, MA) in PBS for 1 h at RT and immune-stained in 3 sequential steps as follows: (i) human anti-CD34 (1:30 dilution, clone QBend10, Beckman Coulter, Brea, CA) overnight at 4 °C followed by incubation with Secondary Rhodamine Red™-X (RhdRx) AffiniPure F (ab′)_2_ Fragment Donkey Anti-Human IgG (H+L) (1:60 dilution, Jackson Immunoresearch Laboratories, Bar Harbor, ME) for 1 h at RT, (ii) human biotinylated-CD43 (1:30 dilution, clone MEM-59, GeneTex, Irvine, CA) overnight at 4 °C followed by incubation with APC-conjugated streptavidin (1:60 dilution, Jackson Immunoresearch Laboratories) at RT for 45 min, and (iii) Alexa Fluor 488-conjugated CD31 (1:30, clone JC/70A, Abcam, Cambridge, MA) overnight at 4 °C followed by DNA (nuclear staining) with DAPI (4′,6-diamidino-2-phenylindole; BD Biosciences) for 2 min.

*Confocal microscopy of immuno-stained fixed organoids* in the ibidi culture dishes (μ-Slide 8 Well, ibidi GmbH, Martinsried, Germany) was performed using a Zeiss 780 confocal microscope (Carl Zeiss Microscopy, Jena, Germany) equipped with a Plan-Apochromat 20x/0.8 NA objective lens. Imaging was via 4 sequential tracks: DAPI fluorescence was imaged using a 405 nm excitation and 415–470 nm emission (displayed in blue), CD31-FITC fluorescence was imaged using 488 nm excitation and 490-555 nm emission (displayed in magenta), CD34-RhdRx fluorescence was imaged using 561 nm excitation and 563–633 nm emission (displayed in red), and CD43-APC fluorescence was imaged using 633 nm excitation and 638–747 nm emission (displayed in yellow). For volume rendering, we collected series of x-y-z images (typically 1 × 1 × 1 μm^3^ voxel size) along the *z*-axis at 2.5 μm intervals over a range of depths (40–120 μm) throughout the whole-mount organoid including large tiled-areas. Representative confocal images were processed by contrast linear stretch only for better visualization.

*Confocal microscopy of viable (live) organoids labeled fluorescently* was conducted using a previously published method with slight modifications [22, 23]. Briefly, 3 FP [Cerulean, EGFP, and tdTomato] or 4FP [Cerulean, EGFP, Venus, and tdTomato] were imaged using a spectral (lambda-λ) scanning (xy-λ)-on the Zeiss 780 spectral detector at 458 nm excitation followed by linear unmixing and 3-channel separation in ZEN software (based on our own recorded single fluorescence proteins emission spectra). For the 4FP, the spectral imaging was performed on the Leica SP8 STED 3X; (Leica Microsystems, Mannheim, Germany), using an HC PL APO CS2 63x/1.40 oil objective lens, a system equipped with a white-light laser tunable from 470 to 670 nm, using 472 nm excitation and recording emission spectra (xy-λ) for single FP-expressing cells as well as 4FP-expressing organoids on a HyD detector with step-size of 10 nm moving in 7.5-nm increments across the entire emission range (from 480 to 700 nm). Recorded spectra were then imported into the dye-database and 4FP images were linearly unmixed in the 4-corresponding channels through Leica LAS-X software 3.5.6 (Leica Microsystems, Mannheim, Germany). Very large z-stacks and tiles were collected, automatically stitched, spectrally unmixed, and processed further using Imaris software to generate 3D-rendered images and rotations-videos.

*Confocal time-lapse experiments* were also performed on the Zeiss 780 confocal system using the 3FP-expressing organoids plated on μ-Dish 35 mm, high dishes (ibidi GmbH) in the organoid differentiation media using the spectral (xy-λ) imaging described above. We imaged for up to 72 h (at 30 min intervals) taking 25 μm z-stacks (xy-λ) and small tiles (or multiple positions) surveilling repeatedly same areas using a × 20 objective, in an environmental chamber maintained at 37 °C and 5% CO_2_, using definite focus routine in ZEN software to maintain imaging stability over long time periods. Images were stitched and linear-unmixed in ZEN software and then exported in Imaris to generate time-lapse movies.

*Two-photon microscopy* imaging was performed using an upright Leica SP8 DIVE (Deep In Vivo Explorer) 2-photon system (Leica Microsystems, Mannheim, Germany). We used in 2-photon mode, for excitation, an infra-red laser InSight X3 from Spectra-Physics dual-beam one fixed 1045-nm line and one tunable line from 680 to 1300 nm. This system has also a second infra-red laser Chameleon Vision II from (Coherent Santa Clara, CA), a pulsed Ti:sapphire laser tunable for excitations from 680 to 1080 nm. Imaging of freshly prepared whole-mount 3D organoids expressing 3FP (Cerulean, EGFP, and tdTomato) was performed using Leica IRAPO L25x/1 W motCorr objective lens (working distance 2.6 mm). We used detection on 4-Tune (tunable) module-all HyDs external non-descanned, tunable detectors (NDDs). In brief, sequential excitation at 1045 nm (InSight laser) was used for Cerulean, second harmonic generated intrinsic signal (SHG) from fibrillar collagen, and tdTomato was combined with a second sequence using excitation at 945 nm (Coherent laser) for EGFP fluorescence. Acquisition (detection) was on all 4 NDDs HyD detectors and their range was set as follows NDD1 (419–480 nm) for Cerulean, NDD2 (517–527 nm) for SHG, NDD3 (528–540 nm) for EGFP, and NDD4 (685–695 nm) for tdTomato, respectively.

For 3D volume rendering, we collected series of x-y-z images (typically 1 × 1 × 1 μm^3^ voxel size) along the *z*-axis at 2.5 μm intervals over a range of depths (40–120 μm) throughout the depth of whole-mount organoid, over large regions using the tile function (Navigator) of the Leica LAS-X software to automatically generate stitched volumes. For 3D renderings and quantitative image analyses, we used Imaris v 9.5.1 software (Bitplane, Zurich, Switzerland).

### Statistical analysis

Statistical analysis was performed by GraphPad Prism Software (v.6.05; GraphPad Software, USA) using one-way analysis of variance and Tukey’s post hoc test. The statistical difference between two groups was evaluated by a two-tailed *t* test. Experiments were performed in triplicate (experimental replicates) except RP-HPLC for globin analysis, and standard errors are represented as error bars. A *p* value of < 0.05 was considered to be statistically significant.

## Results

**Fig. 1:**
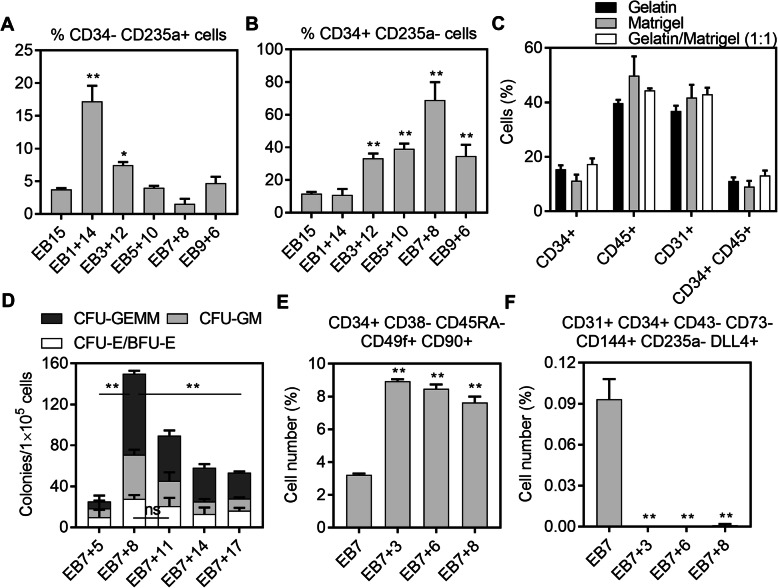
Two-step serum/feeder-free hematopoietic differentiation from human embryonic stem cells (ESCs) displayed high colony-forming unit (CFU) potential and generated a significant portion of hematopoietic stem cell (HSC)-like cells. 7-day embryoid body (EB) maturation followed by 8-day organoid differentiation provided **a** the lowest percentage of primitive (CD34− CD235a+) protocol and **b** the highest percentage of definitive (CD34+ CD235a−) progenitors. **c** Matrigel or gelatin coating did not result in a statistically significant difference in hematopoietic cell emergence. **d** CFU potential, **e** HSC-like, and **f** hemogenic endothelium cell (HE) fraction in different experimental groups (*n* = 3, *p < 0.05, **p < 0.01)

We have previously established a serum- and feeder-dependent definitive HSPC differentiation protocol from human ESCs and iPSCs that can generate erythroid progeny expressing adult globins (γ-globin and β-globin) [14, 24]. We further adapted our protocol to serum-free conditions and demonstrated its clinical relevancy by producing definitive adult globins expressing RBCs from mutation corrected iPSCs generated from fibroblasts from a patient with sickle cell disease (SCD) [12]. To more fully investigate the clinical potential of the protocol, we aimed to eliminate feeders. To do so, we first evaluated differentiating ESC clumps in the organoid differentiation media (Table 1) containing VEGF on plates coated with various coating materials including gelatin, matrigel, retronectin, and fibronectin. However, ESC clumps did not form organoid-like structures and started to detach after 3–5 days of differentiation (data not shown), possibly due to inefficient mesodermal specification of ESCs. Therefore, we combined a widely used EB formation protocol [18] and our organoid differentiation protocol. We first investigated the optimal EB transfer time for a 15-day total HSPC differentiation period. The results revealed that 1-day EB maturation followed by 14-day organoid differentiation (EB1+14) on irradiated OP9 stromal cells produced the highest level of primitive progenitors (CD34− CD235a+, 17.2 ± 2.4%, *p* < 0.01), while all other groups (except the EB3+12 group, *p* < 0.05) were not significantly different (n.s.). The EB7+8 group generated a slightly lower percentage of primitive progenitors (n.s.) compared to the control group, EB15 (Fig. 1a). On the other hand, definitive progenitors (CD34+ CD235a−) were significantly higher in EB3+12, EB5+10, EB7+8, and EB9+6 groups (33.2–69.0%, *p* < 0.01) compared to the EB15 control group (11.2 ± 1.2%) (Fig. 1b). As the EB7+8 group produced the lowest number of primitive and the highest number of definitive progenitors, we selected this protocol for further evaluation.

To adapt the protocol for feeder-free conditions, we transferred 7-day EBs onto gelatin, matrigel, or gelatin/matrigel (1:1) mixture-coated plates and differentiated the EBs for an additional 8 days in organoid differentiation media. Different coating conditions did not result in any statistical difference in hematopoietic cell surface marker expressions including CD34+, CD45+, CD31+, and CD34+ CD45+ (Fig. 1c). To make the protocol more economically feasible, we used gelatin coating for further experiments. In feeder-free conditions, the EB7+8 protocol generated the highest CFU potential (149.8 ± 11.7%, *p* < 0.01), while longer incubation periods resulted in lesser CFUs (Fig. 1d). To further explore the hematopoietic differentiation potential of the optimized protocol, we explored the percentages of HSPC-enriched and HE cell fractions throughout the differentiation period. The HSPC-enriched fraction reached 8.9% at day 10 of hematopoietic differentiation and was slightly reduced at the end of differentiation (7.6%) (Fig. 1e, Supplemental Figure 1). On the other hand, a small fraction of HE cells was detected at the end of EB maturation at day 7 (0.09 ± 0.01%) but the fraction was at almost undetectable levels after this time point (Fig. 1f).

### Organoid differentiation of human ESCs generates definitive erythrocytes

**Fig. 2:**
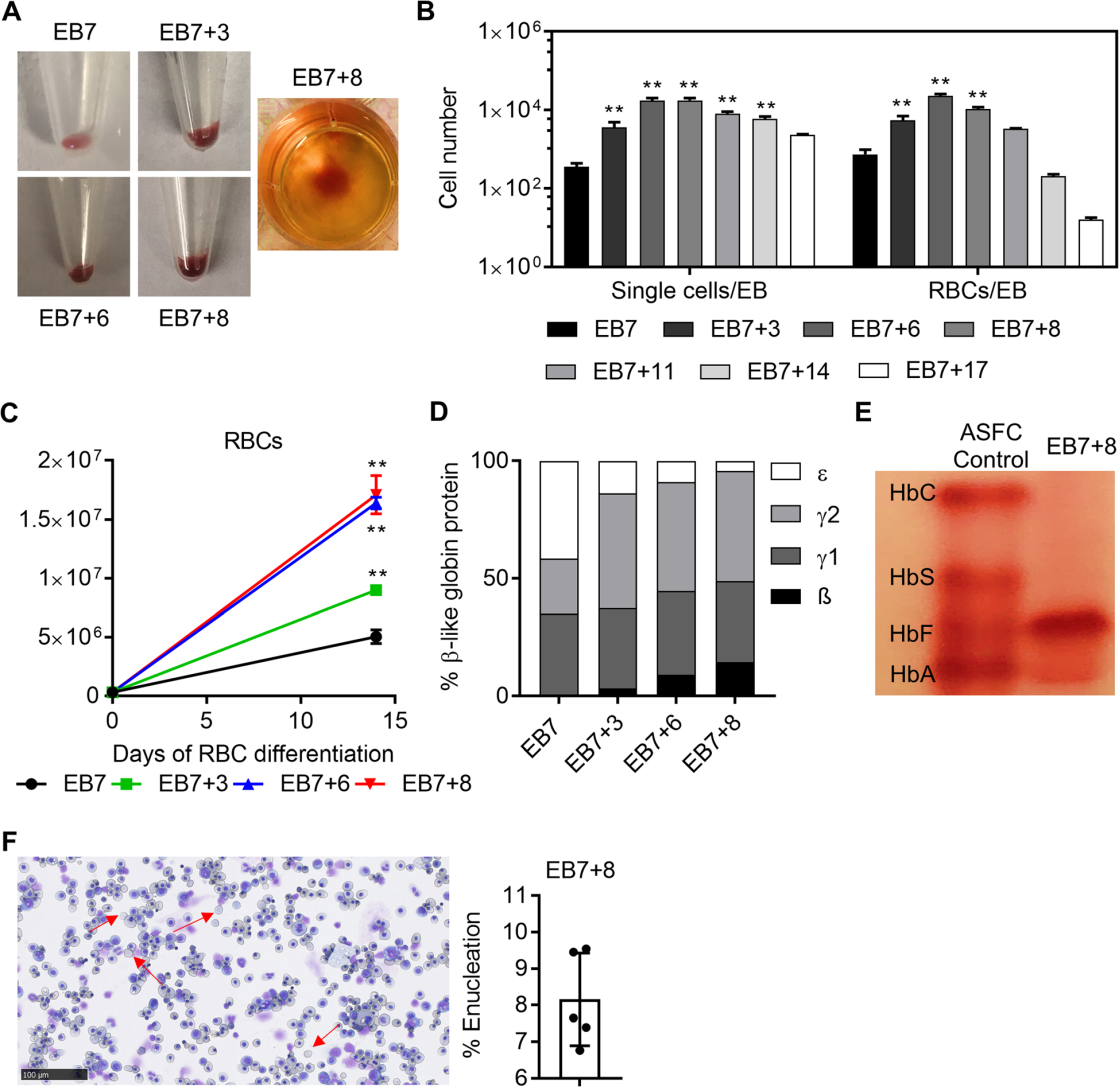
Hematopoietic stem and progenitor cells (HSPCs) from human embryonic stem cells (ESCs) generated definitive red blood cells (RBCs). **a** Hemoglobinization was more evident and robust in later periods of differentiation. **b** Single cells and RBCs per embryoid body (EB). **c** RBC numbers in different experimental conditions throughout RBC differentiation. **d** Globin chain expression by reversed-phase high-performance liquid chromatography (*n* = 1). **e** Hemoglobin electrophoresis and **f** enucleation efficiency for RBCs derived from the EB7+8 group (*n* = 5, **p < 0.01)

To define the primitive/definitive characteristic of HSPCs derived from human ESCs, we first differentiated HSPCs towards erythrocytes and measured definitive globin profiles of the resulting RBCs. Hemoglobinization in the differentiated cells became more evident and robust at later time points of differentiation (Fig. 2a). Single cells derived from EBs were higher in EB7+ (3-14) groups compared to EB7 (*p* < 0.01), while the longer organoid-like differentiation protocol, EB7+17, resulted in lower single-cell yield per EB (Fig. 2b). Similarly, EB7+(3-8) groups produced more RBCs per EB compared to the EB7 group (*p* < 0.01). Although the starting cell number was the same in different groups, RBC proliferation rates in the EB7+3, EB7+6, and EB7+8 groups were significantly higher (*p* < 0.01) (Fig. 2c). RP-HPLC analysis of differentiated RBCs demonstrated that EB7-derived RBCs expressed only γ- and ε-globin (Fig. 2d), while the first detection of definitive β-globin expression occurred in RBCs from the EB7+3-HSPC group (3.2%). Longer organoid differentiation time in HSPC differentiation resulted in higher β-globin expressing RBCs with the highest level noted in the EB7+8 group (14.5%) (Fig. 2d), confirming the definitive potential of HSPCs from human ESCs. Globin expression profile in the EB7+8 group was confirmed with hemoglobin electrophoresis showing robust fetal globin expression and detectable adult globin expression (Fig. 2e). Additionally, differentiated RBCs from the EB7+8 group displayed mostly polychromatic erythroblast stage and 8.1 ± 1.3% of cells underwent successful enucleation (Fig. 2f).

### Human ESC-derived CD34+CD43+ cells display robust CFU potential and generate T-lymphocytes with a broad TCR repertoire

**Fig. 3:**
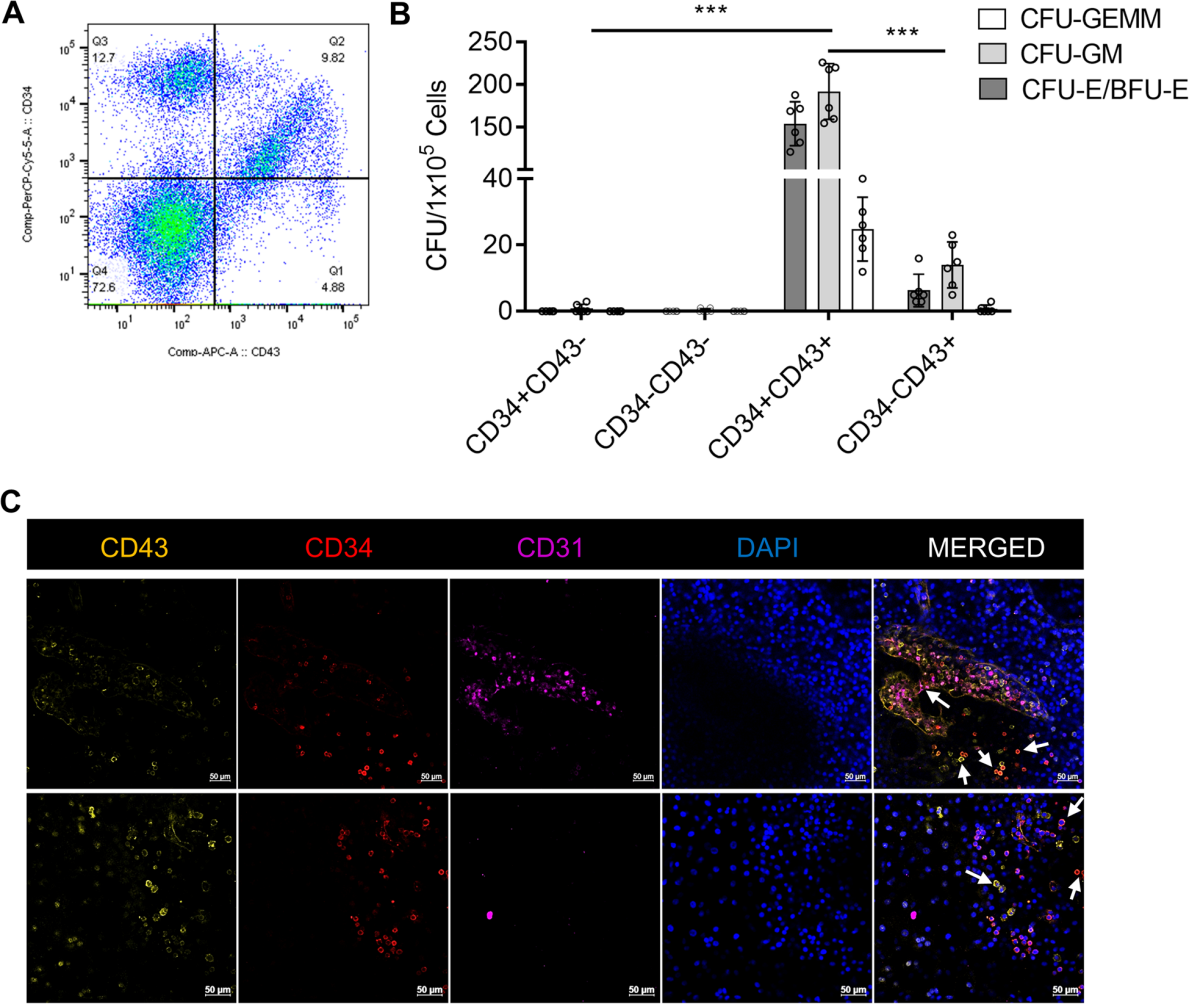
Myeloid and erythroid colonies are strictly restricted to CD43+ cells and co-expression of CD34 markedly enhanced colony-forming unit (CFU) potential. **a** Representative image for sorting strategy for CD34+ and CD43+ cells. **b** CFUs in CD34 and CD43 fractions (*n* = 6, ***p < 0.001). **c** Confocal imaging of organoids stained for CD43, CD34, CD31, and DAPI

Single cells from ESCs were sorted for CD34 and CD43 markers to define CFU potential of different fractions (Fig. 3a). Results revealed that CFU potential is strictly restricted to CD43+ cells. CD34+ CD43+ double-positive cells demonstrated ~ 18-fold CFU potential (370.5 ± 65.7, *p* < 0.01) compared to CD43+ single-positive cells (21.6 ± 11.9) (Fig. 3b), while CD43− fractions did not display robust CFU ability. Next, we imaged antibody-stained organoid-like structures at day 15 of differentiation to locate CD34+ and CD43+ cells’ positioning. Non-attached spherical cells in sac-like structures were mostly positive for CD34/CD43 markers. Single- and double-positive cells in the same sacs indicate different maturation stages of HSPCs (Fig. 3c). Given HSPCs from ESCs displayed definitive characteristics, a close association of CD34+ and CD43+ cells with CD31+ cells indicates the potential emergence of HSPCs from HE through EHT (Fig. 3c).

**Fig. 4:**
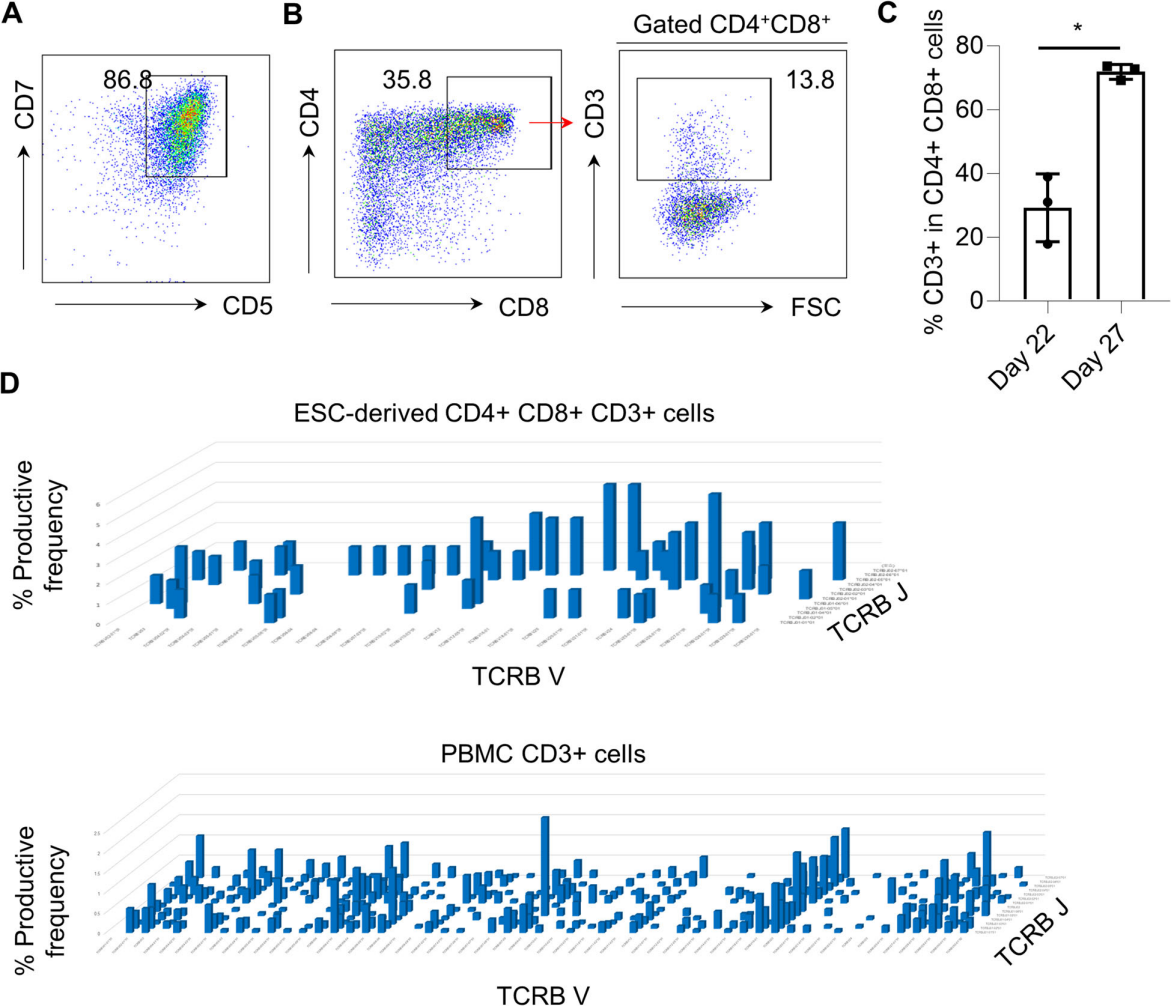
CD34+ CD43+ cells derived from human ESCs generate T-lymphocytes with a broad T cell receptor (TCR) repertoire. **a** Representative flow cytometry analysis of CD5 and CD7 expression in ESC-derived CD34+ CD43+ cells after 22 days of T cell differentiation on OP9/DLL1 co-culture. **b** Representative flow cytometry analysis of CD4, CD8, and CD3 expression in ESC-derived CD34+ CD43+ cells at 22 days of T cell differentiation on OP9/DLL1 co-culture. **c** The frequency of CD3+ cells in CD4+CD8+ double-positive T cells on day 22 and day 27. Values represent mean ± SD (*n* = 3). **d** TCR-Vβ deep sequencing showed the diversity of rearranged TCR genes in ESC-derived CD4+ CD8+ CD3+ T cells at day 27 of differentiation

As CD34+ CD43+ cells from organoid-like structures displayed myeloid and erythroid lineage potential with definitive potential, we sought to explore whether the 2-step differentiation system is able to produce hematopoietic progenitors with T-lymphoid potential. To test this, we used the commonly applied OP9/DLL1 co-culture system, providing extrinsic Notch signaling to induce the differentiation of HSPCs into T cell lineage [20]. CD34+ CD43+ cells harvested from the EB7+8 group were co-cultured with OP9/DLL1 stromal cells and supplemented with FLT-3L, IL-7, and SCF. By day 22 of T cell differentiation, most ESC-derived CD34+ CD43+ cells expressed CD5 and CD7 T progenitor markers (Fig. 4a), and more mature CD4+ CD8+ double-positive (DP) T cells also could be detected (Fig. 4b). From day 22 to day 27 of T cell differentiation, the CD3 expression on CD4+ CD8+ DP T cells increased dramatically that suggests extracellular presentation of the TCR complex (Fig. 4b, c). To further probe the potential of ESC-derived CD34+ CD43+ cells to generate a broad TCR repertoire, we compared expression of the TCR-Vβ repertoire in ESC-derived T cells and peripheral blood mononuclear cells (PBMC) by DNA sequencing. TCR-Vβ deep sequencing demonstrated that ESC-derived CD4+CD8+CD3+ on day 27 showed a broad TCR repertoire similar to CD3+ cells isolated from PBMCs (Fig. 4d, Supplemental Table 1), indicating that ESC-derived HSPCs retain the capacity to produce lymphocytes ex vivo.

### Spherical cells in sacs derive from rare single progenitors

To have more insight into cellular dynamics in the organoid-like structures, we aimed to label undifferentiated ESCs with different FPs to be imaged during hematopoietic differentiation. In our preliminary studies, we transduced ESCs with lentiviral vectors expressing Cerulean, EGFP, Venus, or tdTomato. After 3 days of lentiviral transduction, cells were sorted for high FP-expressing cells. However, at 5–7 days of culture in undifferentiated conditions, most of the sorted ESCs lost their FP expression (data not shown). Thus, to have stable FP expression, we applied *AAVS1* safe harbor targeting using CRISPR/Cas9 and donor template consisting of an FP sequence. After 3 day culture of RNP electroporated cells in undifferentiated conditions, a small number of cells (~ 1–2%) were positive under microscopic evaluation. Positive cells were picked from bulk cultures and grown on separate plates. Then, pre-confluent cells (at ~ 60–70% confluency) were sorted by high FP-expressing cells (Supplemental Figure 2) and cryopreserved for later use. Sorted cells did not silence FP expression during maintenance (up to 10 passages tested) and differentiation. Separately labeled human ESCs were mixed during EB formation in two groups: 3FP (Cerulean, EGFP, and tdTomato) and 4FP (Cerulean, EGFP, Venus, and tdTomato).

**Fig. 5:**
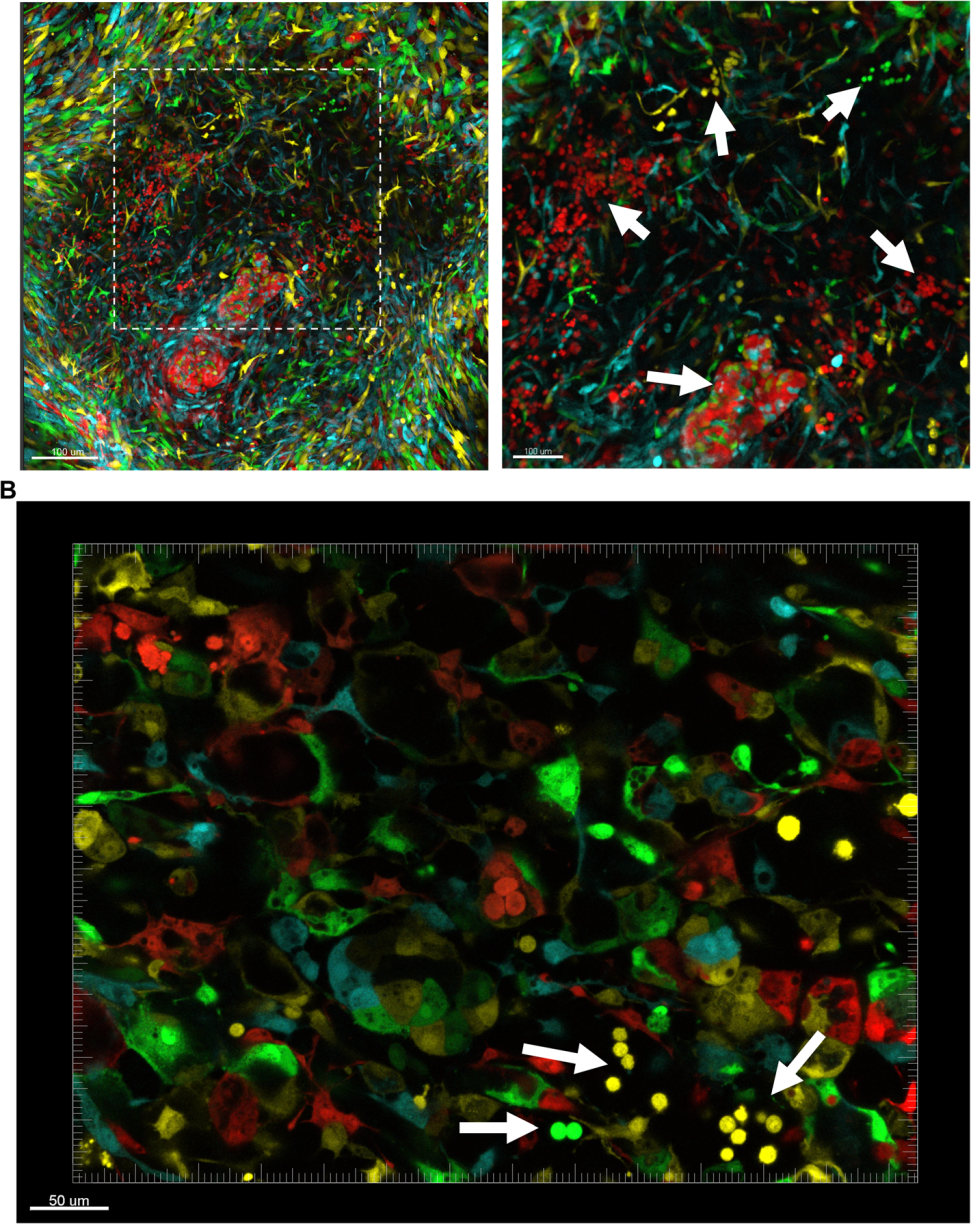
Spherical non-attached cells cluster by color, indicating differentiation from rare progenitors that underwent serial proliferation. **a** Confocal imaging of 3-color (Cerulean, EGFP, and tdTomato) and **b** 4-color (Cerulean, EGFP, Venus, tdTomato) organoids at day 15 of differentiation. White arrows indicates spherical non-attached cell clusters


For confocal imaging, emission spectra for each FP were done to set the configuration (Supplemental Figure 3A). To confirm that specific channels did not overlap with each other, HeLa cells were transduced with lentiviral vectors (LeGo vectors) expressing respective FPs and imaged using confocal microscopy. Imaging of single FP-expressing HeLa cells revealed the absence of cross-talk between channels (Supplemental Figure 3B). After transferring fluorescently labeled EBs onto gelatin-coated plates, EBs attached and started to spread from the center. Spreading cells produced a mixture of the spindle-shaped (fibroblast-like) and elongated/hexagonal-shaped (endothelial-like) cells around the center of organoid-like structures (Supplemental Figure 4A). After day 10 of differentiation, spherical non-attached cells started to emerge and accumulated inside the sacs in the center of the structure (Supplemental Figure 4A). At day 15 of differentiation, flow cytometry analysis of organoid single cells demonstrated no statistically significant difference in the Cerulean, EGFP, and tdTomato fractions (Supplemental Figure 4B), supporting the absence of FP silencing during differentiation and survival advantage of FP-labeled ESCs. Interestingly, confocal imaging of the structures depicted that single color spherical non-attached cells clustered in different locations of the structure (Supplemental Figure 4C, Fig. 5a, b, Supplemental Video 1, 2, 3, 4, 5), proposing that cells most likely emerged from single rare HE cells followed by a series of cell divisions rather than HSPC specification of different HE cells within the same spherical cell cluster. Spherical non-attached cells emerged at the borders of the center of the structure where spindle-shaped and endothelial-like elongated cells started to spread out (Supplemental Figure 4C). The edges of the organoids were mostly positive for CD34-expressing elongated/hexagonal cells, suggesting that the spreading cells have an endothelial origin (Supplemental Figure 4D). Furthermore, several tube-like structures in the organoids were noted, suggesting high endothelial cell activity (Fig. 5b). Next, we applied two-photon confocal imaging to observe the collagen structure inside FP-labeled organoid structures. The results revealed that organoids display collagen-rich structures, and localize and interact with non-attached spherical cells (Supplemental Figure 4E). Sac structures that accumulated visible spherical non-attached cells consisted of a complex superficial dense collagen fiber network (Supplemental Figure 4E, Supplemental Video 6, 7).

**Additional file 7: Supplemental Video 2.** Time-lapse imaging of a 3 fluorescence protein [cyan (Cerulean), green (EGFP), and red (tdTomato)] organoid from 12 to 15 days (72 h at 30 min intervals) of hematopoietic differentiation.


**Additional file 9: Supplemental Video 4.** 3D reconstruction of z-stacks-tile merged confocal microscopy images of a 3 fluorescence protein [cyan (Cerulean), green (EGFP), and red (tdTomato)] organoid at day 15 of hematopoietic differentiation.

## Discussion

We previously demonstrated that organoid differentiation of human ESCs/iPSCs on mouse stromal cells (C3H10T1/2 or OP9) generate definitive HSPCs capable of producing adult globin-expressing RBCs [12, 14, 24]. To establish a clinically relevant protocol and eliminate feeder dependency, we coupled spin EB and organoid differentiation protocols. After optimization of EB maturation and organoid differentiation time, we generated definitive HSPCs from human ESCs that produce T cells with broad TCR repertoire (comparable to PBMC T cells) and significant levels of definitive adult β-globin (14.5%) expressing RBCs with improved enucleation rate compared to our previously published protocol [14] (Table 2). In the human embryo, the second wave of hematopoiesis in the yolk sac and third wave in the AGM region produce definitive HSPCs from a specific set of HE cells through the EHT process [25]. Mice transplantation studies revealed that very few numbers of AGM-HSPCs have the capacity to reconstitute the whole hematopoietic system in conditioned recipients [26]. On the other hand, while yolk sac definitive HSPCs can produce T cells and RBCs with adult globin expression, they still lack engraftment potential [27, 28]. Although our differentiation system could provide T-lymphocytes with a broad TCR repertoire and RBCs expressing β- and γ-globins, mouse transplantation assays are required to explore the engraftment potential of human ESC-derived HSPCs. Alternatively, definitive HSPCs from iPSCs generated using this approach could be used in the clinic for transfusion purposes after required purification/selection strategies and good manufacturing practice-grade cell production standards are applied.

**Table 2:** Comparison of previously published ES-Sac and the current serum/feeder-free differentiation protocols

	Relative cost of differentiation protocols	RBC differentiation time (Days)	Enucleation rate in RBCs	β-globin protein expression in RBCs
ES-Sac (Previous feeder-dependent methods) [12, 14]	$$	32	1.40%	4%
Serum/feeder-free organoid differentiation method	$$$	32	8%	14.5%

Immunocompromised mouse transplantation models are the only available tools to evaluate the engraftment potential of de novo generated HSPCs. However, this method is costly, time-consuming, and requires expertise in animal handling. Establishment of ex vivo models for evaluating engraftment capacity of HSPCs from ESCs/iPSCs would increase the speed and cost of screening. To do so, engraftable HSPCs must be identified, fully characterized, and a greater understanding of HE cell specification and EHT processes are warranted. One way to accomplish these goals would be to define cell surface markers that discriminate different waves of HSPCs. CD34, a transmembrane phosphoglycoprotein, is routinely used in the clinic to select and enrich engrafting HSCs from mobilized peripheral blood or intact bone marrow samples [29]. However, expression of CD34 in various cell types, particularly in stromal precursor cells [30] and vascular endothelial cells [31], would limit its usage in multicellular organoids for HSPC detection and enrichment. As a previously appreciated hematopoietic cell specification marker [32, 33], here, we confirmed CD43, leukosialin, for the enrichment of definitive HSPCs from human ESC-generated organoids. CFU potential was restricted to CD43-expressing cells suggesting CD43 is required for hematopoietic specification, with an ~ 18-fold greater CFU capacity when double-positive (CD34+ CD43+) cells are compared to only CD43+-expressing cells. Given adult mobilized CD34 cells co-express CD43 [34] (Supplemental Figure 5), CD43 cell enrichment should also be accompanied in search of definitive HSPCs or engraftable HSCs from pluripotent stem cells. In support of our findings, a recent study conducting single-cell analysis on HSPC differentiated human iPSCs identified CD34+ CD43+ fraction as an HSC-like cell-enriched population, CD34− CD43+ fraction as more differentiated hematopoietic progenitors, and CD34+ CD43− cells as vascular endothelial cells [35]. Additionally, imaging of antibody-stained organoid-like structures revealed clusters of spherical non-attached cells in the sac structures with single- and double-positive expressions for CD34 and CD43, suggesting that HSPC populations are more heterogeneous at different differentiation stages and multipotent progenitors continue to differentiate after their emergence.

As complex and dynamic interactions of HSPCs with their neighboring cells and matrix proteins are critical for stem cell emergence and maintenance [36], recent advances in imaging technology offer an effective tool to elucidate the stem cell niche. To have more insight into cellular dynamics in the organoid structures, we labeled undifferentiated ESCs with different FPs and mixed them during EB formation. Confocal imaging of the organoids demonstrated single color spherical cell clusters in the border of the organoid center, supporting HSPC specification from rare HE cells followed by serial proliferation cycles, as well as indicating the importance of the positioning of the ESCs in the organoid structure for an efficient EHT process. Similarly, very few cells with high proliferation capacity emerge as HSCs from HE cells in zebrafish embryos [16] and the dorsal aorta of the AGM region in the human embryos [37, 38].

The extracellular matrix (ECM) is a critical support protein within the embryo and interacts with several hematopoietic factors including IL-3 and SCF to provide close interactions between stromal cells, HSCs, and cytokines [39, 40]. A recent pre-print article reported that a vital component of ECM proteins, *hapln1b*, mediates kit ligand b (*kitlgb*)-kit b (*kitb*) interactions and is required for HSC specification from HE in the zebrafish embryo [41]. In our system, two-photon confocal microscopy imaging demonstrated that spherical non-attached cells are enveloped in multilayer collagen fiber networks within the sac structures of organoids. Within these structures, denser matrix networks may enhance interactions between cells and cytokines, hence, more efficient HSPC generation, yet this hypothesis needs to be addressed. Additional studies characterizing full ECM structure in the AGM region and HSPC generating organoids, and their interactions with the niche would further improve ex vivo differentiation protocols.

## Conclusion

Here, we demonstrate an optimized two-step serum/feeder-free hematopoietic differentiation protocol from human ESCs that generated definitive RBCs expressing adult globin and T cells with a broad TCR repertoire. CFU potential was restricted to CD43-expressing cells suggesting CD43 is required for hematopoietic specification, with myeloid and erythroid colony-forming progenitors detected mainly in the CD34+ CD43+ fraction of organoid cells. Imaging of the organoids confirmed the emergence of potentially engraftable, definitive progenitors as described in the third wave of hematopoiesis, visibly emerging from HE cells through EHT. This serum/feeder-free two-step hematopoietic differentiation protocol from pluripotent stem cells aids in the establishment of clinical-grade ex vivo protocols for the generation of definitive HSPCs and/or potentially engraftable HSC production for transplantation studies.

## Supplementary Information

**Additional file 1: Supplemental Figure 1.** Representative image for sorting strategy for CD34+ CD38− CD45RA− CD49f+ CD90+ (Hematopoietic stem cell-like cells) population.

**Additional file 2: Supplemental Figure 2.** Flow cytometry sorting strategy for fluorescently labeled undifferentiated human embryonic stem cells.

**Additional file 3: Supplemental Figure 3. (A)** Emission spectra of Cerulean, EGFP, Venus, and tdTomato. **(A)** Imaging of HeLa cells transduced with lentiviral vectors (LeGo vectors) expressing different fluorescent proteins.

**Additional file 4: Supplemental Figure 4.** Single color spherical cells clustered at the borders of organoid center and accumulated in collagen fiber formed sacs. **(A**) Development of hematopoietic cell generating organoids after transfer 7-day old embryoid body (EB) onto gelatin-coated plates. (**B**) Fluorescently labeled fractions in organoids at day 15 of differentiation. (**C**) Confocal imaging of 3-color labeled organoids. White arrows indicates spherical non-attached cell clusters. (**D**) CD34 staining of the edge of an organoid. (**E**) Two-photon microscopy imaging of 3-FPs (Cerulean, EGFP, tdTomato) -expressing organoids.

**Additional file 5: Supplemental Figure 5.** Flow cytometry panels for CD34 and CD43 staining of CD34-enriched peripheral blood plerixafor mobilized cells.

**Additional file 6: Supplemental Video 1.** 3D reconstruction of z-stacks-tile merged confocal microscopy images of a 4 fluorescence protein [cyan (Cerulean), green (EGFP), yellow (Venus), and red (tdTomato)] organoid at day 15 of hematopoietic differentiation.

**Additional file 8: Supplemental Video 3.** Time-lapse imaging of a 3 fluorescence protein [cyan (Cerulean), green (EGFP), and red (tdTomato)] organoid from 12 to 15 days (72 h at 30 min intervals) of hematopoietic differentiation.

**Additional file 10: Supplemental Video 5.** 3D reconstruction of z-stacks-tile merged confocal microscopy images of a 3 fluorescence protein [cyan (Cerulean), green (EGFP), and red (tdTomato)] organoid at day 15 of hematopoietic differentiation.

**Additional file 11: Supplemental Video 6** 3D reconstruction of z-stacks-tile merged confocal microscopy images of a 3 fluorescence protein [cyan (Cerulean), green (EGFP), and red (tdTomato)] organoid enmeshed in collagen fibers (SHG, white) at day 15 of hematopoietic differentiation.

**Additional file 12: Supplemental Video 7.** 3D reconstruction of z-stacks-tile merged confocal microscopy images of a 3 fluorescence protein [cyan (Cerulean), green (EGFP), and red (tdTomato)] organoid enmeshed in collagen fibers (SHG, white) at day 15 of hematopoietic differentiation.

**Additional file 13: Supplemental Table 1.** Raw sequencing data for CD3 cells derived from CD34+ CD43+ generated from human embryonic stem cells (hESCs) and peripheral blood mononuclear cells (PBMCs).

## Data Availability

All data generated or analyzed during this study are included in this manuscript and its supplementary information files.

## Abbreviations

HSCs: Hematopoietic stem cells
HSPCs: Hematopoietic stem and progenitor cells
ESCs: Embryonic stem cells
iPSCs: Induced pluripotent stem cells
EB: Embryoid body
TCR: T cell receptor
RBCs: Red blood cells
HE: Hemogenic endothelium
AGM: Aorta gonad-mesonephros
EHT: Endothelial-to-hematopoietic transition
BMP-4: Bone morphogenic protein-4
bFGF: Basic fibroblast growth factor
SFM: Serum-free media
VEGF: Vascular endothelial growth factor
PBS: Phosphate buffer solution
CFU: Colony-forming unit
FLT-3: FMS-like tyrosine kinase 3 ligand
TPO: Thrombopoietin
EPO: Erythropoietin
FBS: Fetal bovine serum
BSA: Bovine serum albumin
TFA: Trifluoroacetic acid
FP: Fluorescent protein
PFA: Paraformaldehyde
SCD: Sickle cell disease
