# Correction to: Definitive hematopoietic stem/progenitor cells from human embryonic stem cells through serum/feeder-free organoid-induced differentiation

**DOI:** 10.1186/s13287-020-02082-y

**Published:** 2020-12-17

**Authors:** Selami Demirci, Juan J. Haro-Mora, Alexis Leonard, Claire Drysdale, Daniela Malide, Keyvan Keyvanfar, Khaled Essawi, Raul Vizcardo, Naritaka Tamaoki, Nicholas P. Restifo, John F. Tisdale, Naoya Uchida

**Affiliations:** 1 Sickle Cell Branch, National Heart Lung and Blood Institute (NHLBI)/National Institute of Diabetes and Digestive and Kidney Diseases, National Institutes of Health (NIH), 9000 Rockville Pike, Bldg. 10, 9N112, Bethesda, MD 20892 USA; 2 Light Microscopy Core Facility, NHLBI, NIH, Bethesda, MD USA; 3 Clinical Flow Core Facility, NHLBI, NIH, Bethesda, MD USA; 4 National Cancer Institute, Center for Cancer Research, NIH, Bethesda, MD USA

**Correction to: Stem Cell Res Ther (2020) 11:493**

**https://doi.org/10.1186/s13287-020-02019-5**

The original article [1] contains an error in the captions of Additional Files 11 and 12.

In each caption, the word ‘confocal’ should be replaced by ‘two-photon’.
