# Supplementary figures and images for: Definitive hematopoietic stem/progenitor cells from human embryonic stem cells through serum/feeder-free organoid-induced differentiation
Source: Stem Cell Res Ther. 2020 Nov 24;11:493. doi: 10.1186/s13287-020-02019-5 (PMC7688003; doi:10.1186/s13287-020-02019-5)

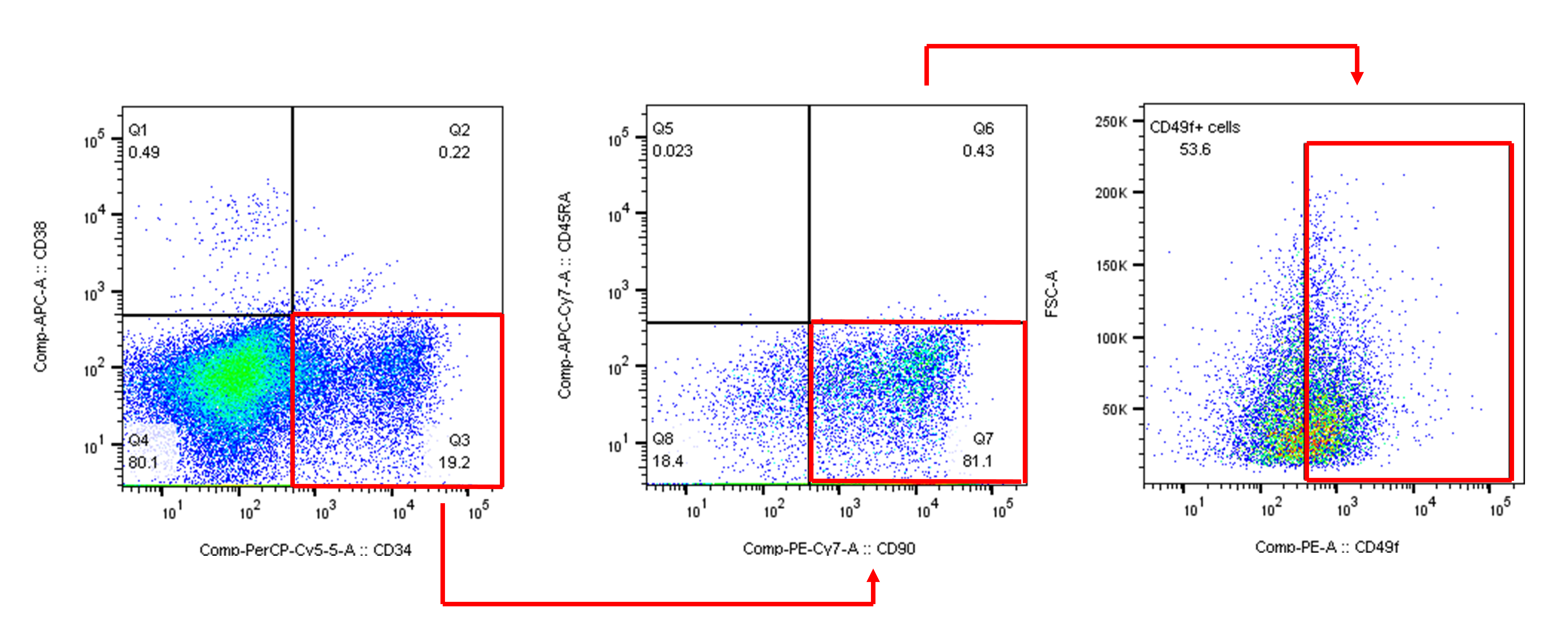

Supplement: Supplementary file 3 — Additional file 1: Supplemental Figure 1. Representative image for sorting strategy for CD34+ CD38− CD45RA− CD49f+ CD90+ (Hematopoietic stem cell-like cells) population. [file 13287_2020_2019_MOESM1_ESM.tif]

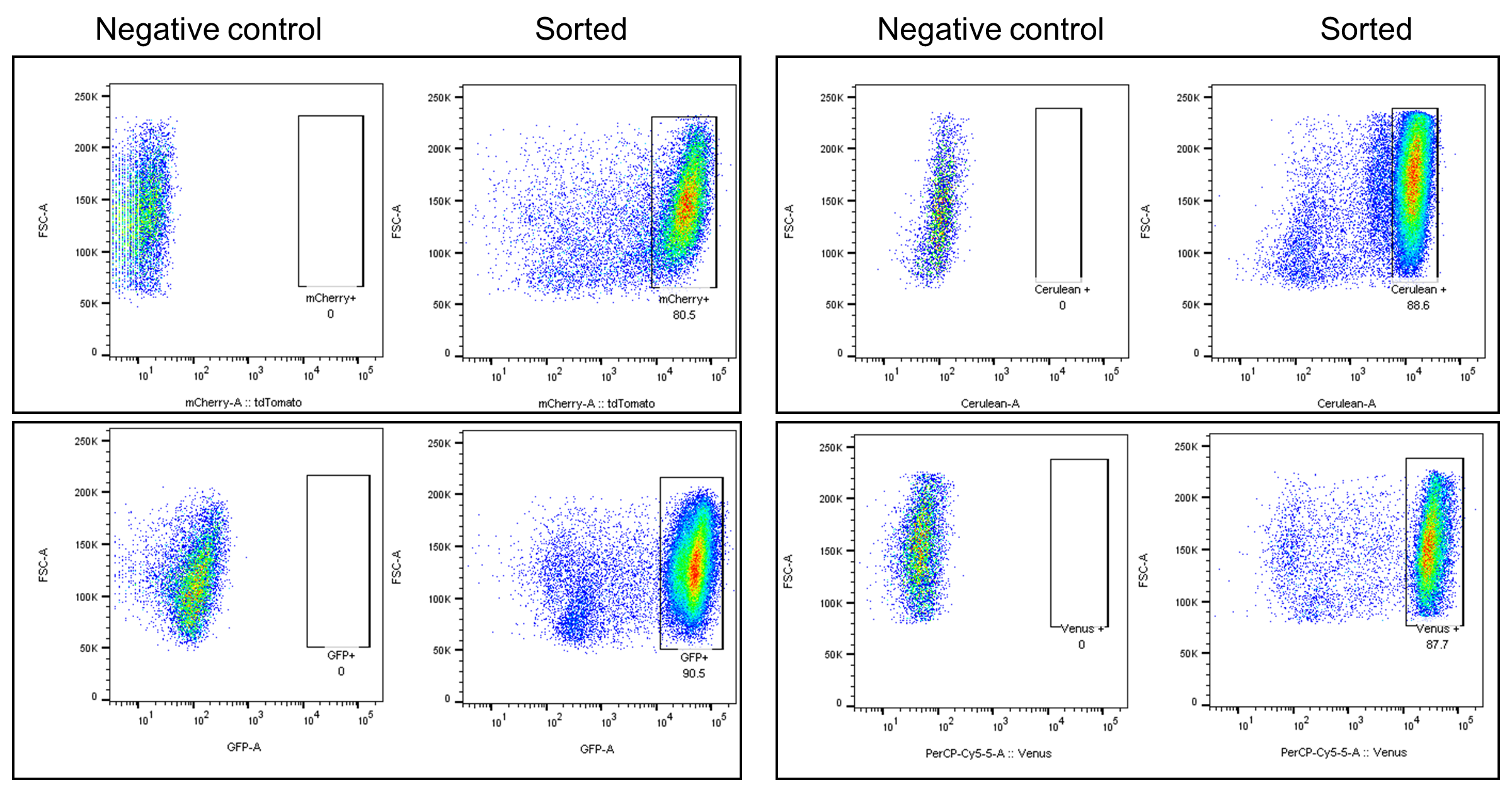

Supplement: Supplementary file 4 — Additional file 2: Supplemental Figure 2. Flow cytometry sorting strategy for fluorescently labeled undifferentiated human embryonic stem cells. [file 13287_2020_2019_MOESM2_ESM.tif]

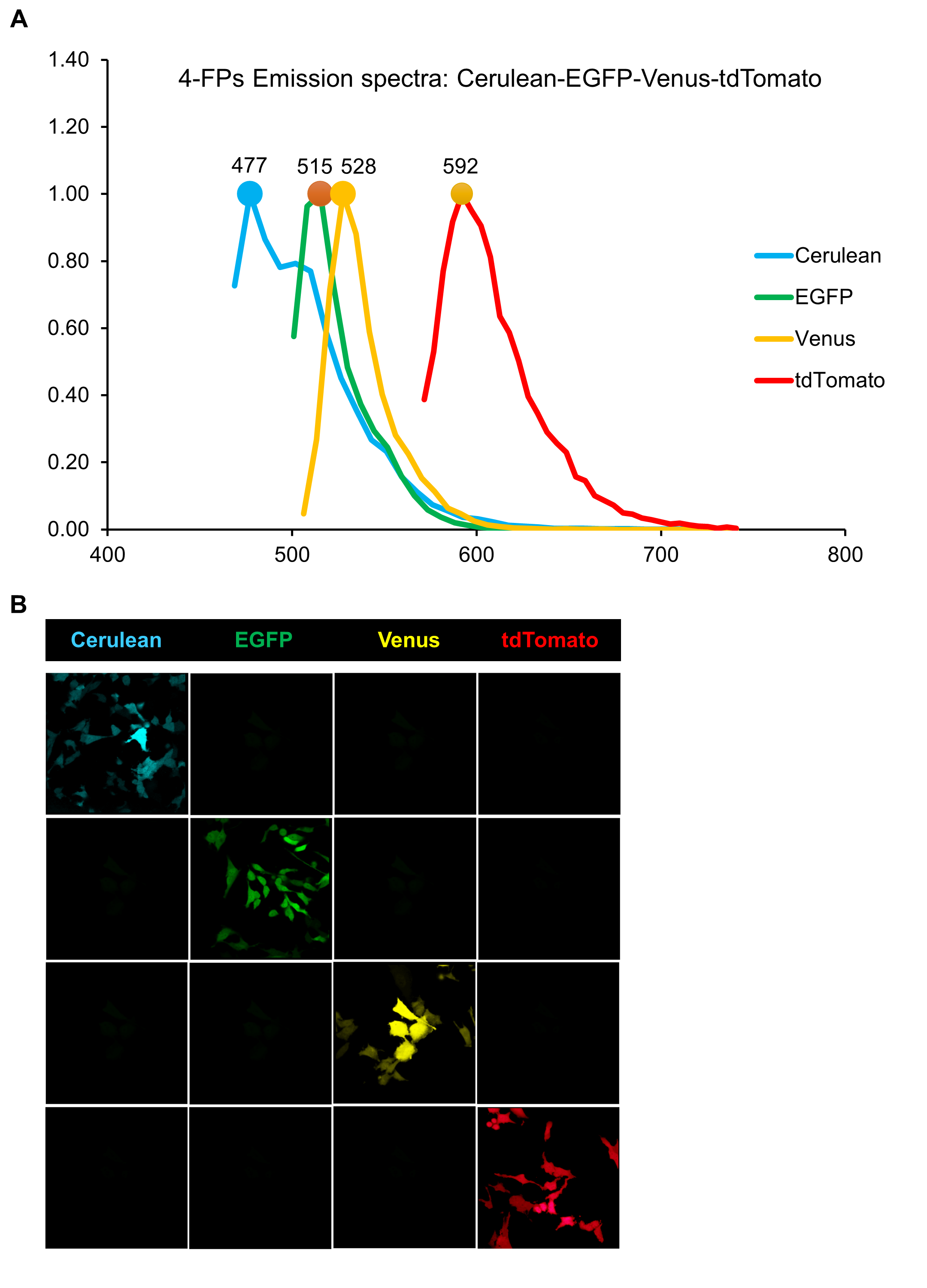

Supplement: Supplementary file 5 — Additional file 3: Supplemental Figure 3. (A) Emission spectra of Cerulean, EGFP, Venus, and tdTomato. (A) Imaging of HeLa cells transduced with lentiviral vectors (LeGo vectors) expressing different fluorescent proteins. [file 13287_2020_2019_MOESM3_ESM.tif]

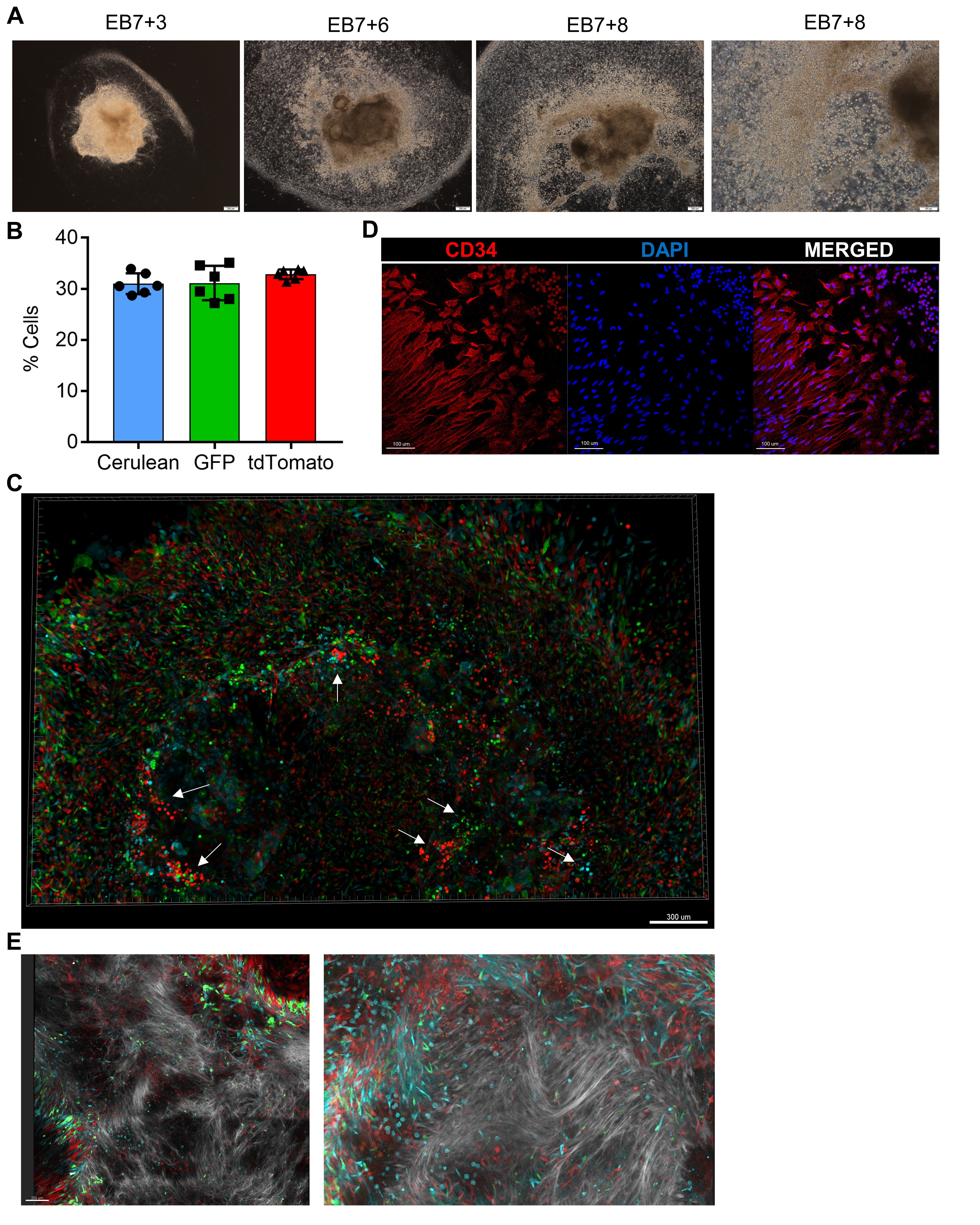

Supplement: Supplementary file 6 — Additional file 4: Supplemental Figure 4. Single color spherical cells clustered at the borders of organoid center and accumulated in collagen fiber formed sacs. (A) Development of hematopoietic cell generating organoids after transfer 7-day old embryoid body (EB) onto gelatin-coated plates. (B) Fluorescently labeled fractions in organoids at day 15 of differentiation. (C) Confocal imaging of 3-color labeled organoids. White arrows indicates spherical non-attached cell clusters. (D) CD34 staining of the edge of an organoid. (E) Two-photon microscopy imaging of 3-FPs (Cerulean, EGFP, tdTomato) -expressing organoids. [file 13287_2020_2019_MOESM4_ESM.tif]

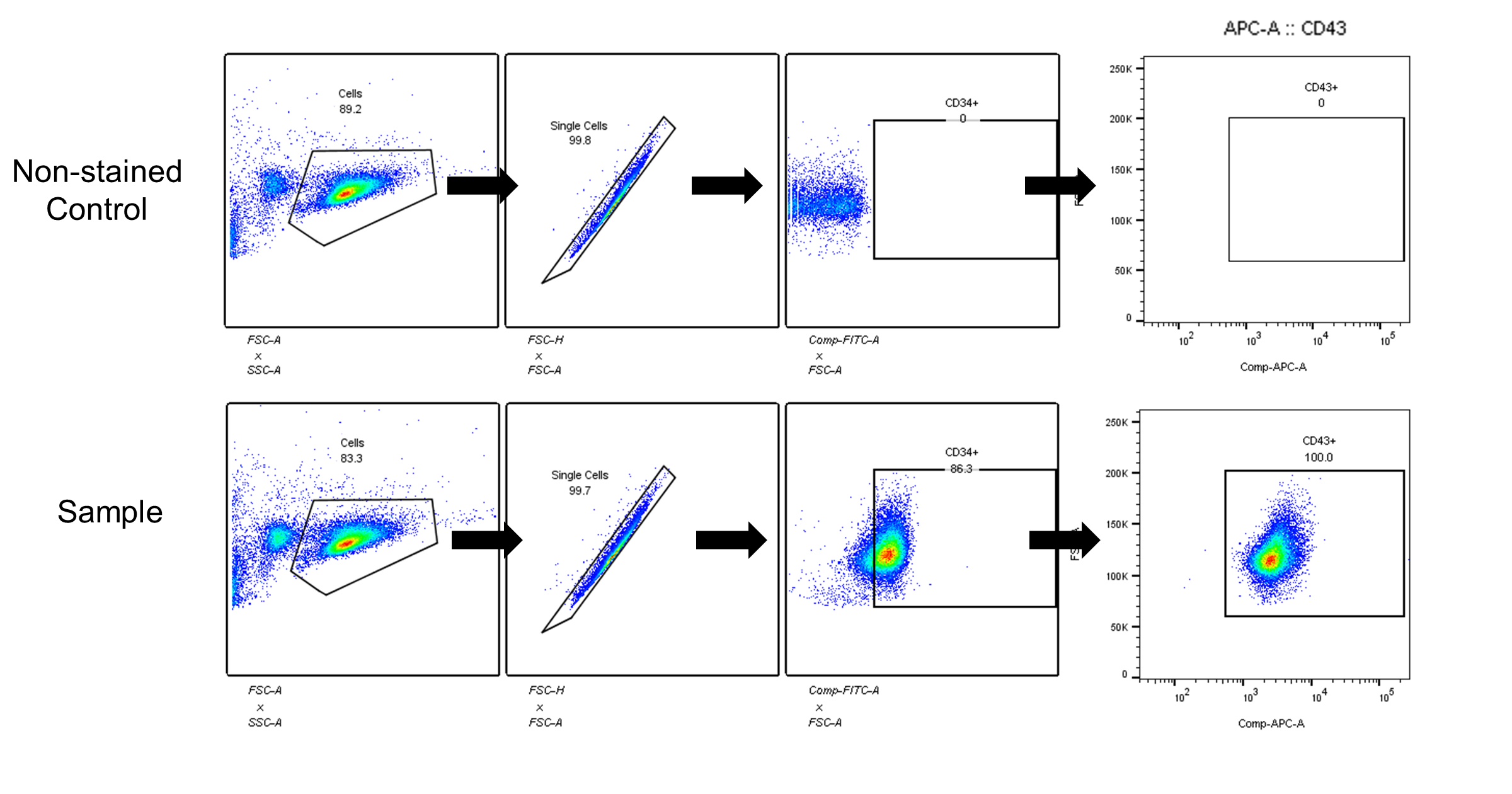

Supplement: Supplementary file 7 — Additional file 5: Supplemental Figure 5. Flow cytometry panels for CD34 and CD43 staining of CD34-enriched peripheral blood plerixafor mobilized cells. [file 13287_2020_2019_MOESM5_ESM.tif]
